# Structure deformation of indium oxide from nanoparticles into nanostructured polycrystalline films by *in situ* thermal radiation treatment

**DOI:** 10.1186/1556-276X-8-428

**Published:** 2013-10-17

**Authors:** Su Kong Chong, Siti Nur Azieani Azizan, Kee Wah Chan, Hong-Quan Nguyen, Wee Siong Chiu, Zarina Aspanut, Chang Fu Dee, Saadah Abdul Rahman

**Affiliations:** 1 Low Dimensional Materials Research Centre, Department of Physics, University of Malaya, Kuala Lumpur 50603, Malaysia; 2 Department of Materials Science and Engineering, National Chiao Tung University, Hsinchu 30010, Taiwan; 3 Institute of Microengineering and Nanoelectronics (IMEN), Universiti Kebangsaan Malaysia (UKM), Bangi, Selangor 43600, Malaysia

**Keywords:** Indium oxide, Nanoparticles, Structure deformation, Optical, Electrical

## Abstract

A microstructure deformation of indium oxide (In_2_O_3_) nanoparticles by an *in situ* thermal radiation treatment in nitrous oxide plasma was investigated. The In_2_O_3_ nanoparticles were completely transformed into nanostructured In_2_O_3_ films upon 10 min of treatment time. The treated In_2_O_3_ nanoparticle sample showed improvement in crystallinity while maintaining a large surface area of nanostructure morphology. The direct transition optical absorption at higher photon energy and the electrical conductivity of the In_2_O_3_ nanoparticles were significantly enhanced by the treatment.

## Background

Indium oxide (In_2_O_3_), known as an n-type, wide-band gap (2.9 to 3.1 eV) semiconductor [1,2], is of great interest for diverse technological applications in nanoelectronics and optoelectronics [3]. Zero-dimensional In_2_O_3_ nanoparticles (NPs), with a variety of tunable morphologies ranging from octahedra, hexagons, cubes, to pyramids, are beneficial as building blocks for indium oxide-based or hybrid transistors [4]. Their remarkably large surface-to-volume ratio and good stability have made them promising materials in gas sensors/biosensors [5,6], photocatalysis [7], photoelectrochemical cells [8], and ultraviolet photodetectors [9,10]. Despite the advantages of using this material, In_2_O_3_ NP-based devices usually encounter several deficiencies, for instance, low conductivity and poor adhesion. This could decrease the efficiency and stability of the devices. One of the reasons for the low conductivity of In_2_O_3_ NP-based devices is due to the weak interconnection between each NP [11,12]. In this case, the carrier transportation between the In_2_O_3_ NPs is inefficient where charge carriers might be lost at the interface due to recombination or charge delocalization. Meanwhile, the In_2_O_3_ NP coating is usually not adhesive, thus making it easier to be scratched from the substrate. Hence, in order to solve these problems, it is crucial to improve the microstructure arrangement of the In_2_O_3_ NPs.

Several methods such as annealing and plasma treatments have been introduced to improve the structural and electrical properties of In_2_O_3_ nanostructures [13-15]. A previous report [13] showed an increase in photoconductivity of undoped In_2_O_3_ thin films to about 10^2^ (Ω cm)^−1^ by using a two-step thermal annealing method at an optimum temperature of ≤500°C. More recent research on femtosecond laser annealing of In_2_O_3_ nanowire transistors revealed significant improvements in device performance owing to the reduction in interfacial traps by using the treatment [14]. On the other hand, oxygen plasma treatment [15] serves as an alternative treatment method to improve the surface contact of tin-doped In_2_O_3_ for light-emitting devices. By combining rapid thermal annealing and nitrous oxide (N_2_O) plasma treatment, Remashan et al. [16] demonstrated almost two orders of increment in off current and on/off current ratios of zinc oxide thin film transistors.

A significant effort has been devoted to the advancement in synthesis and fabrication of In_2_O_3_ NPs using a variety of techniques including laser ablation, electron beam evaporation, chemical vapor deposition (CVD), pulsed laser deposition, sol-gel, and thermolysis [17,18]. Of those, CVD is capable of high yield production and good crystallinity of In_2_O_3_ NPs [19]. The In_2_O_3_ NPs synthesized by this method typically have a higher purity level compared to those synthesized by wet chemical methods as the deposition is done under a certain vacuum level. In addition, the CVD-grown In_2_O_3_ NPs are usually oxygen deficient and have better conductivity than the homogenous stoichiometric In_2_O_3_[8]. In this study, a novel deposition of In_2_O_3_ NPs using a modified plasma-assisted hot-wire chemical vapor deposition (PA-HWCVD) system is reported. The deposition was done by evaporating the bulk indium wire in a nitrous oxide plasma environment. The vaporized indium atoms were oxidized by the oxidizing agents, then forming In_2_O_3_ NPs on the substrates. We demonstrate an effective way to improve the structural, optical, and electrical properties of the In_2_O_3_ NPs by introducing an *in situ* thermal radiation treatment under an oxidizing agent plasma condition. Compared to the previously reported treatment methods [13-16], the proposed method offers a cost-effective, single-step deposition process to perform treatment on the as-deposited samples. In addition to surface treatment, this method can also be used to control the microstructure morphology and crystallinity of the In_2_O_3_ nanostructures to suit desired applications.

## Methods

In_2_O_3_ NPs were deposited on a quartz substrate using a home-built PA-HWCVD system (Additional file 1: Figure S1). Indium wire (purity 99.999%) with a diameter of 0.5 mm and a length of approximately 2 mm was used as indium source. Tantalum filament coils were used for indium evaporation. The filament coils were preheated in H_2_ ambient at approximately 1,500°C for 10 min to remove the contamination before being used for deposition. The distance of the electrode and the filament with the substrate is fixed at 5 and 3 cm, respectively. The quartz substrate was heated to 300°C in vacuum (10^−3^ mbar) before starting deposition. Evaporation process was then carried out at a filament temperature of approximately 1,200°C in a N_2_O plasma environment. The rf power density for the N_2_O plasma generation is fixed at 1.273 W cm^−2^. The deposition pressure and N_2_O gas flow rate were controlled at 1 mbar and 60 sccm, respectively. For thermal radiation treatment, the temperature of the filament increased rapidly to about 1,800°C for 7 to 10 min after complete evaporation of the indium wire by the hot filament. The N_2_O plasma generation was terminated at 5 min after the evaporation process or the thermal treatment process.

A Hitachi SU 8000 field emission scanning electron microscope (FESEM; Hitachi, Tokyo, Japan) attached with an EDAX Apollo XL SDD detector energy dispersive X-ray (EDX) spectroscope (EDAX Inc., Mahwah, NJ, USA) was utilized to perform surface morphology study and chemical composition analysis of the samples. Structural properties of the samples were studied using a Siemens D5000 X-ray diffractometer (Siemens Corporation, New York, NY, USA) and a Renishaw InVia photoluminescence/Raman spectrometer (Renishaw, Wotton-under-Edge, UK). X-ray diffraction (XRD) patterns were obtained using Cu Kα radiation at a glazing angle of 5°, and Raman spectra were recorded under an excitation of argon laser source with a wavelength of 514 nm. Photoluminescence (PL) properties of the samples were examined using a Renishaw InVia PL/Raman spectrometer under an excitation of He-Cd laser at 325 nm. High-resolution transmission electron microscopy (HRTEM) micrographs of the samples were taken using a JEOL 2010 HRTEM (JEOL Ltd., Tokyo, Japan). A PerkinElmer Lambda 750 UV/VIS/NIR spectrometer (PerkinElmer, Waltham, MA, USA) was employed to obtain the optical transmission, reflectance, and absorbance of the samples. The optical reflectance spectra were measured at an incident angle of 45° to the samples. Electrical properties of the samples were studied using a Keithley Source Measure Unit 236 (Keithley Instruments, Inc., Cleveland, OH, USA) for current-voltage (*I*-*V*) measurement. Prior to the *I*-*V* measurement, gold electrodes (in circular shape, diameter of about 2 mm) were evaporated on top of the sample using a thermal evaporator. The distance between two consecutive electrodes was fixed at 2 mm.

## Results and discussion

**Figure 1:**
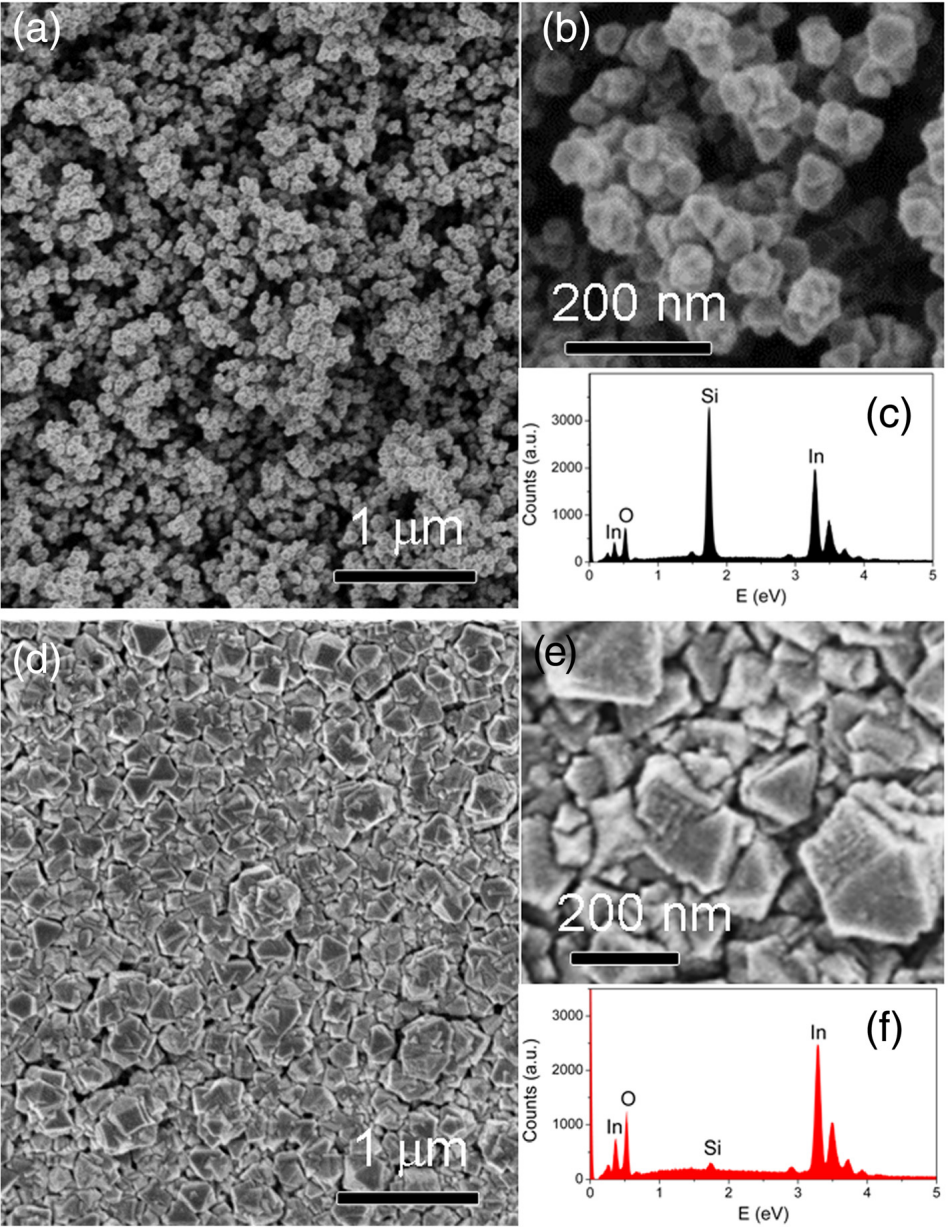
**FESEM images and EDX spectra.** FESEM images of **(a**, **b)** as-grown In_2_O_3_ NPs and **(d**, **e)** thermal radiation-treated In_2_O_3_ NPs. **(c**, **f)** EDX spectra of the as-grown In_2_O_3_ NPs and thermal radiation-treated In_2_O_3_ NPs, respectively.

Figure 1a shows the FESEM images of the In_2_O_3_ NPs formed by the evaporation of In wires in a N_2_O plasma environment. A high density of NPs with an average size of approximately 40 ± 9 nm was found to be randomly distributed on the quartz substrate. A magnified FESEM image (Figure 1b) reveals the appearance of the NPs. Structures with different numbered facets (three, four, five, six, and eight faces) corresponding to triangular, rhombohedral, pentagonal, hexagonal, and octahedral shapes, respectively, can be recognized from the sample. These structures indicate that the In_2_O_3_ NPs formed are in crystalline state. The observed In and O signals from the energy-dispersive X-ray (EDX) spectrum (Figure 1c) confirm the composition of the In_2_O_3_ NP. The Si signal that appeared in the EDX spectrum originated from the quartz substrate. The color of the In_2_O_3_ NPs changed from white to yellowish upon thermal radiation treatment (Additional file 1: Figure S2). The films appear to be more transparent after the treatment. The FESEM image depicted in Figure 1d reveals a compact nanostructured film for the sample after undergoing thermal radiation treatment. The sizes of the nanostructures vary largely from 60 to 300 nm. Meanwhile, we observed that the nanostructures mainly consist of shapes with fewer facets which are triangular or rhombohedral (Figure 1e). The EDX spectrum taken from the nanostructured films (Figure 1f) showed high signals of In and O, reflecting high purity of the nanostructured In_2_O_3_ films formed by this technique. The signal of the substrate (Si) was largely suppressed due to the closely packed structure of the In_2_O_3_ film, which limited the emission of X-ray from the substrate atoms after the thermal radiation treatment.

**Figure 2:**
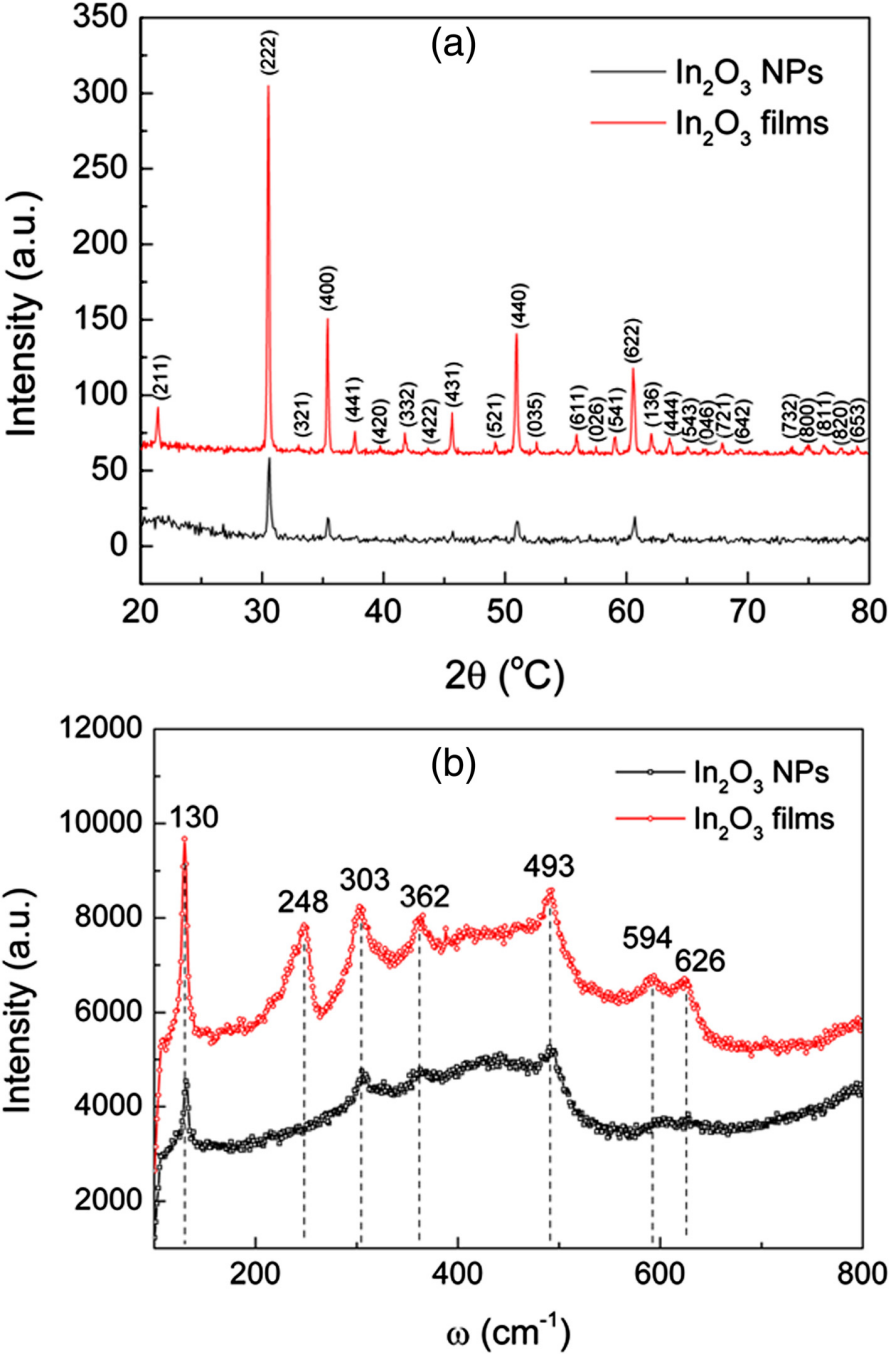
**XRD patterns and Raman spectra. (a)** XRD patterns and **(b)** Raman spectra of In_2_O_3_ NPs and nanostructured In_2_O_3_ films.

Figure 2a shows the XRD patterns of the In_2_O_3_ NPs and the nanostructured In_2_O_3_ films formed after thermal treatment. The crystalline peaks are well indexed to body-centered cubic (bcc) In_2_O_3_ (JCPDS 76-0152). The absence of the In crystalline peak infers the complete oxidation of the In wire in N_2_O plasma. Thus, highly crystalline structures of In_2_O_3_ with a tendency to form a (222) crystal plane were obtained. The thermal radiation treatment improved the crystallinity of the In_2_O_3_ structure. The appearance of a more In_2_O_3_-related crystalline peak in the XRD pattern indicates a polycrystalline structure, forming the nanostructured In_2_O_3_ films. Crystalline sizes calculated from the In_2_O_3_(222) crystalline peak using the Scherrer formula [20] are 33.8 ± 0.1 nm for the In_2_O_3_ NPs and 43.2 ± 0.1 nm for the nanostructured In_2_O_3_ films. The size of the crystalline In_2_O_3_ NP is close to the measurement taken by FESEM (approximately 40 ± 9 nm), which evidently indicates a single-crystalline structure of the In_2_O_3_ NPs. The size of the crystalline nanostructured In_2_O_3_ film is relatively small compared to the size of the nanostructures (60 to 300 nm). Therefore, the nanostructured In_2_O_3_ film apparently consists of polycrystalline structures with an average crystal size of about 43 nm.

The structural properties of the In_2_O_3_ NPs and nanostructured In_2_O_3_ films were further confirmed by Raman spectra. Consistent with XRD analysis, the Raman spectra also provided evidence of the bcc In_2_O_3_. The observed seven Raman peaks located at 130, 248, 303, 362, 493, 594, and 626 cm^−1^ are assigned to the phonon vibration modes of the bcc In_2_O_3_[21]. The Raman peak of 248 cm^−1^ which was only detected by the highly oriented In_2_O_3_ nanostructure was presumably highly dependent on the orientation of the NPs [22]. Thus, it is usually insignificant in the Raman spectrum of randomly distributed In_2_O_3_ NPs [23]. In addition, PL spectra of the untreated In_2_O_3_ NPs and treated nanostructured In_2_O_3_ films are presented in Additional file 1: Figure S3 to provide a qualitative study on the structure defect of the In_2_O_3_ nanostructures. A broad orange-reddish emission centered at about 610 and about 660 nm was observed in all samples. This emission is normally attributed to the defect emission due to oxygen deficiencies [24] or the intrinsic defects related to oxygen [25]. The suppression of defect-related emission of In_2_O_3_ is correlated to the reconstruction of defect structures and improvement in crystallinity of In_2_O_3_ structures [26] by thermal radiation treatment.

**Figure 3:**
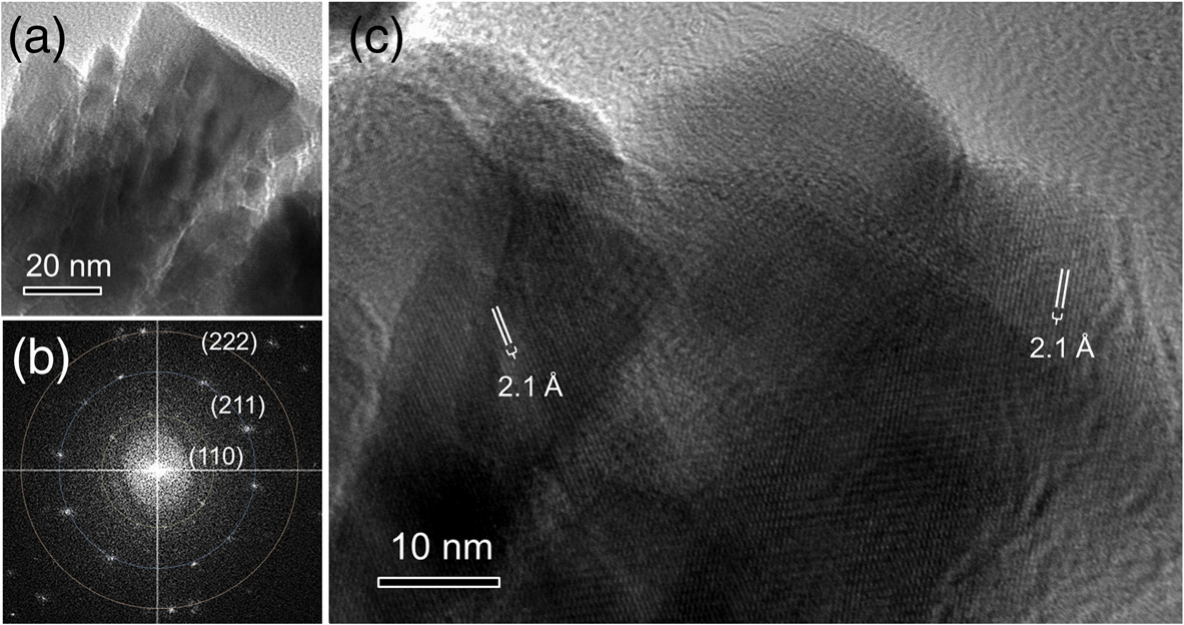
**TEM, FFT, and HRTEM. (a)** TEM micrograph, **(b)** FFT electron diffraction pattern, and **(c)** HRTEM micrograph of the nanostructured In_2_O_3_ films.

HRTEM analysis of the nanostructured In_2_O_3_ films is presented in Figure 3. The TEM micrograph of the nanostructured In_2_O_3_ after thermal radiation treatment (Figure 3a) shows the agglomeration of the In_2_O_3_ NPs to form compact structures. The bundles of In_2_O_3_ formed by stacked In_2_O_3_ nano/microcrystallites can be clearly observed in the figure. Fast Fourier transform (FFT) pattern (Figure 3b) consists of paired bright spots due to the crystalline structure of the individual In_2_O_3_ NPs. The paired spots create diffraction rings indicating a polycrystalline nature of the nanostructured In_2_O_3_ films, which is consistent with the XRD analysis. HRTEM investigation on the individual NPs reveals a single-crystalline In_2_O_3_ structure regardless of their shapes (Additional file 1: Figure S4). Meanwhile, the HRTEM micrograph of the In_2_O_3_ nanostructures treated with thermal radiation (Figure 3c) reveals multiple crystal orientations which provide the evidence of the crystal grains and bundles bonded by the In_2_O_3_ NPs.

**Figure 4:**
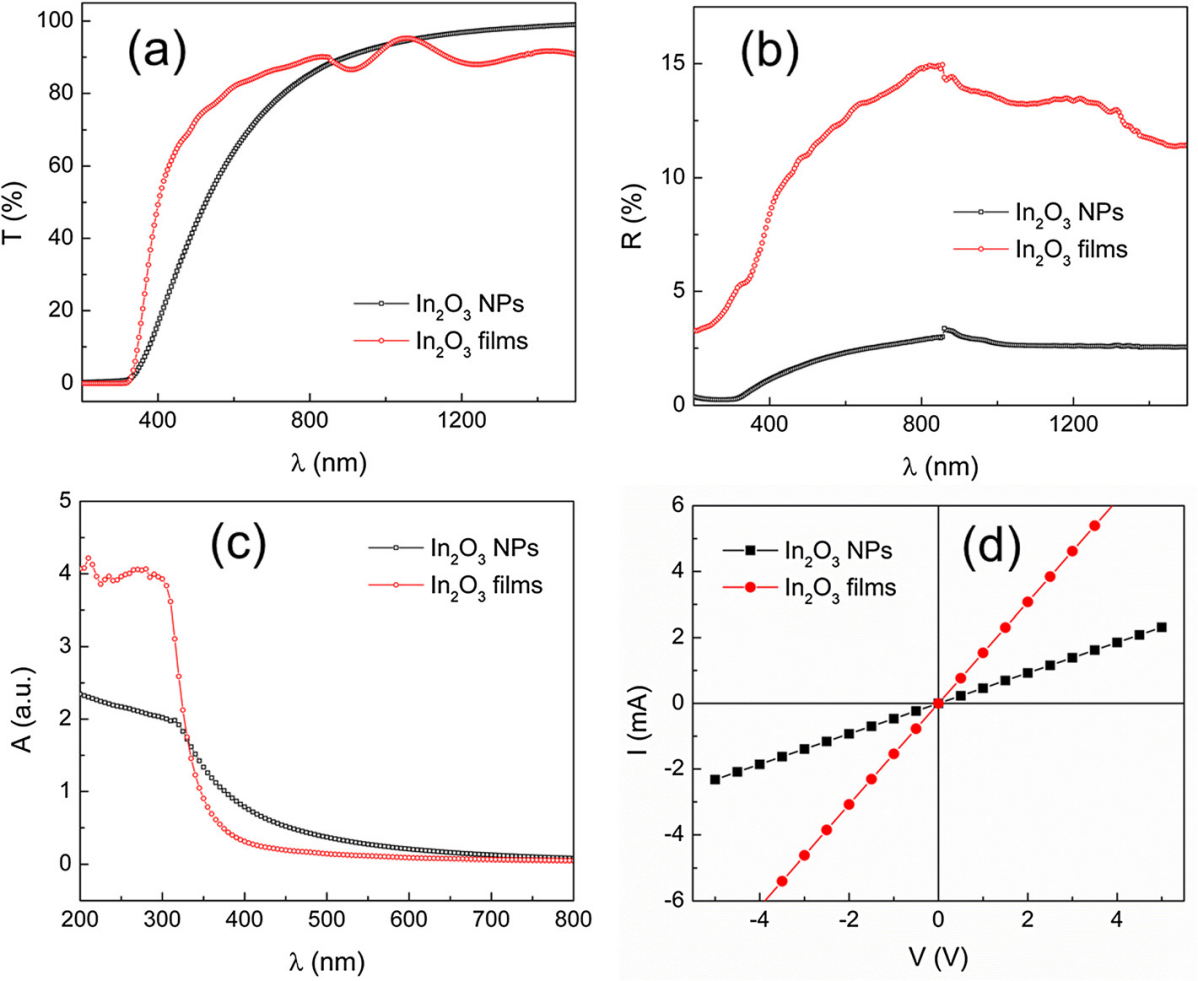
**Optical spectra and *I*-*V *plots.** Optical **(a)** transmission, **(b)** reflectance, and **(c)** absorbance spectra and **(d)***I*-*V* plots of In_2_O_3_ NPs and nanostructured In_2_O_3_ films.

The optical and electrical properties of the In_2_O_3_ NPs and the nanostructured In_2_O_3_ films were also studied. Figure 4a shows the optical transmission (*T*) spectra of both the In_2_O_3_ NPs and nanostructured films. The In_2_O_3_ NPs showed a high *T* of >90% at the NIR region (*λ* > 850 nm). The *T* gradually decreased with the reduction of *λ* in the visible spectral region. For the nanostructured In_2_O_3_ films, the *T* remained greater than 80% at a spectral region of *λ* > 550 nm, while it abruptly decreased to zero at *λ* = 330 nm. Both the *T* spectra of the In_2_O_3_ NPs and nanostructured film coincide at about the same absorption edge (approximately 330 nm), which indicates that there was not much modification of the optical energy gap (*E*_opt_) for the NPs and film structures. Tauc plots for the In_2_O_3_ NPs and nanostructured In_2_O_3_ films are shown in Additional file 1: Figure S5. The *E*_opt_ of the In_2_O_3_ NPs and nanostructured films measured from the Tauc plots were 3.4 ± 0.1 and 3.6 ± 0.1 eV, respectively. Meanwhile, the Tauc plots of In_2_O_3_ NPs and nanostructured films reveal low-energy tails at 2.6 ± 0.1 and 3.0 ± 0.1 eV, respectively, which represent their fundamental band gap (*E*_g_) [2]. The red shift of the *E*_opt_ and *E*_g_ of In_2_O_3_ NPs can be due to the defect in the energy levels formed by the oxygen vacancy in the nanosized In_2_O_3_ crystals [27]. The *E*_g_ value of the In_2_O_3_ nanostructures is closer to the theoretically predicted band gap of bcc In_2_O_3_ (2.9 to 3.1 eV) [1,2] after undergoing a thermal radiation treatment. The lower *T* of In_2_O_3_ NPs in the visible region is attributed to the large surface-to-volume ratio of the structure of the NPs compared to more compact nanostructured films. The large surface area resulted in the total internal reflection between the interlayer of the NPs, effectively trapping the incident photons within the samples. This may also indicate an antireflection behavior for the In_2_O_3_ NP due to its high photon absorption. The optical reflectance (*R*) spectra (Figure 4b) of In_2_O_3_ NPs and nanostructured films are in accordance with this assumption. The *R* of the In_2_O_3_ NPs is <4% within the spectral region of 200 to 1,500 nm, which is about four times lower than that of the nanostructured In_2_O_3_ films.

The optical absorption properties of the In_2_O_3_ NPs and the nanostructured In_2_O_3_ films were further analyzed according to their absorbance (*A*) spectra as shown in Figure 4c. Two spectral regions can be recognized from the *A* spectra. At the visible region (*λ* > 350 nm), the *A* of the In_2_O_3_ NPs was greater than that of the nanostructured In_2_O_3_ films due to the larger surface-to-volume ratio of the NPs, which was previously discussed. Conversely, the *A* of the nanostructured In_2_O_3_ films was about one time greater than that of the In_2_O_3_ NPs at the UV region (*λ* < 350 nm), where the incident photon energy was greater than the *E*_opt_ of In_2_O_3_. The photon absorption at the high-energy (>*E*_opt_) region is attributed to the direct transition of In_2_O_3_[28]. The nanostructured In_2_O_3_ films formed after the thermal treatment process possessed higher crystallinity and more compact structures compared to the In_2_O_3_ NPs. Thus, they can effectively absorb the incident photon during the photon interaction.

*I*-*V* plots of the In_2_O_3_ NPs and nanostructured In_2_O_3_ films are shown in Figure 4d. The increase in slope for the nanostructured In_2_O_3_ films indicates an enhancement in the conductance of the In_2_O_3_. This can be explained by the improvement in the interconnection between the nanostructures of In_2_O_3_ as shown in the FESEM images which thereby improves the charge mobility of the In_2_O_3_ structures. Moreover, the conductivity of the In_2_O_3_ nanostructures is also strongly related to surface-adsorbed oxygen molecules [29]. Upon exposure to air, the electrons in In_2_O_3_ nanostructures will transfer to the surface of the nanostructures and ionize the oxygen source from the air to form an oxygen surface layer. This process creates an electron depletion layer, thus reducing the conductivity of the In_2_O_3_ nanostructures. The large surface-to-volume ratio of the untreated In_2_O_3_ NPs indicates higher resistance compared to the treated nanostructured In_2_O_3_ films due to the significant amount of oxygen molecules bonded to the surface of the NPs which generated a broader electron depletion layer. Resistivity values calculated from the *I*-*V* curves were 4.3 × 10^−2^ and 1.3 × 10^−2^ Ω cm for the In_2_O_3_ NPs and nanostructured In_2_O_3_ films, respectively. The resistivity value of the treated In_2_O_3_ nanostructures is smaller than the reported value for the undoped In_2_O_3_ films (about 5 × 10^−2^ Ω cm) [30].

**Figure 5:**
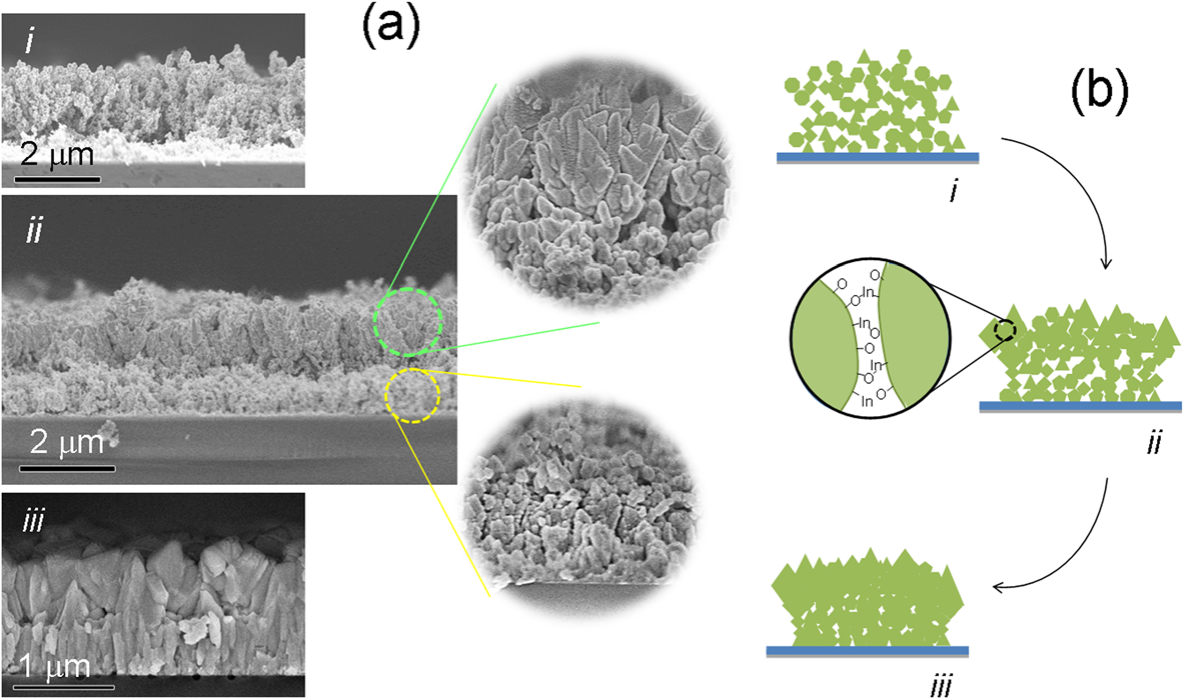
**Mechanism for the evolution of In**_**2**_**O**_**3 **_**NPs to nanostructured In**_**2**_**O**_**3 **_**films. (a)** Cross-sectional FESEM images of In_2_O_3_ NPs (i) without and with (ii) 7 and (iii) 10 min of thermal radiation treatment. The magnified FESEM images from the top and bottom layers of the bilayer nanostructured polycrystalline In_2_O_3_ films in (ii) are shown on the right-hand side of (ii). **(b)** Schematic of the structure deformation of the In_2_O_3_ NPs (i) into the nanostructured In_2_O_3_ films (ii, iii) upon thermal radiation treatment.

The characterizations above demonstrated that by changing their microstructure arrangement through the *in situ* thermal radiation treatment process in N_2_O plasma, there was an improvement in the crystallinity and optical and electrical properties of the In_2_O_3_ NPs. In order to understand the microstructure deformation process, the cross-sectional FESEM images of the untreated and thermally treated In_2_O_3_ NPs were analyzed as shown in Figure 5a. The untreated sample (Figure 5a(i)) showed a random orientation of the In_2_O_3_ NPs on the quartz substrate. The thermal radiation treatment on the In_2_O_3_ NPs (Figure 5a(ii)) subsequently separates the cross section into two layers with different morphologies. A magnified view of the upper layer revealed the stacking of the NPs between each other, forming larger bundles of In_2_O_3_ nanostructures. The In_2_O_3_ bundles were apparently formed by the agglomeration of the In_2_O_3_ NPs due to the thermal treatment. This layer was eventually turned into larger-sized (Figure 5a(iii)). The lower layer was mainly comprised of the In_2_O_3_ NPs, as shown in the magnified image of Figure 5a(ii). However, the NPs seem to be reorganized vertically from the substrate. An increase in the thermal radiation treatment time resulted in the formation of uniform, rod-like structures in the layer between the substrate and pyramid In_2_O_3_ grains (Figure 5a(iii)).

A mechanism for the deformation of the In_2_O_3_ NP structure into the bilayer nanostructured In_2_O_3_ films was thus proposed and illustrated in Figure 5b. In the upper layer (approximately 1 μm), the In_2_O_3_ NPs were expected to be exposed directly to the thermal radiation and plasma treatment. The discharged N_2_O vapors formed large quantities of excited O* species. The thermal radiation from the hot filament supplied extra heat to the O* to form energetic O* species. As the energetic O* species reached the surface of the In_2_O_3_ NPs, they were able to adsorb into the In dangling bonds or to extract the O atoms from the weak In-O bonds. This process activated the surface of the In_2_O_3_ NPs by leaving extra In- and O-free bonds. The closest surface between two NPs had a tendency to form In-O covalent bonds by sharing free electrons, thus resulting in the agglomeration of the In_2_O_3_ NPs. From a thermodynamic consideration, the nanostructures with fewer facets are usually more stable due to their lower surface energy [31]. Thus, in our case, the In_2_O_3_ NPs stacked up into bundles and eventually formed pyramids or cube-like In_2_O_3_ grains with the least number of faces. The transition of structures from octahedra to cubes and further to pyramids as preferred by the In_2_O_3_ nanostructures was confirmed by the planar-view FESEM as shown in Additional file 1: Figure S6a-c.

The microstructure deformation process for the bottom layer is slightly different from that for the top layer. The In_2_O_3_ NP agglomeration on the top layer created coverage for the NPs beneath them. Thus, the exposure of the In_2_O_3_ NPs to the N_2_O plasma was assumed to be negligible in this region. Heat transferred from the upper to the lower layer of the In_2_O_3_ NPs provided excessive energy for the reconstruction of the structure of the NPs. The NPs confined between the upper layer and substrate had enough space to reorganize to their preferred shapes. According to the surface energy of In_2_O_3_, *γ*{111} <*γ*{100} <*γ*{110}, the {111} plane possesses the lowest surface energy [32]. From the HRTEM analysis (Additional file 1: Figure S4), most of the In_2_O_3_ NPs were showing the (222) crystallographic plane. The NPs tended to reorganize in order to maximize the more stable {111} plane. One possible way was to arrange them vertically along the [100] or [110] direction with the lateral facet in the {111} plane. This explains the vertical alignment of the In_2_O_3_ NPs to form a rod-like structure in the bottom layer of the sample.

## Conclusions

In summary, we demonstrated an effective method to enhance the crystal structure, direct transition absorption, and electrical conductivity of In_2_O_3_ NPs by introducing a thermal radiation treatment. We attributed these enhancements to the improvement in the microstructure of the In_2_O_3_ NPs to the nanostructured In_2_O_3_ films. This tractable and tunable microstructure deformation process is useful in a variety of In_2_O_3_-related technologies.

## Competing interests

The authors declare that they have no competing interests.

## Authors' contributions

SK, KW, and SNA carried out the experimental parts on sample preparation and characterization. HQ and WS carried out the TEM and HRTEM measurements. SK drafted the manuscript. ZA, CF, and SA participated in the analysis and discussion and revised the manuscript. All authors read and approved the final manuscript.

## Supplementary Material

Additional file 1 **Supplementary information. Figure S1.** Schematic diagram and real time photographs of our home-built PA-HWCVD system. **Figure S2.** Photograph of the In_2_O_3_ NPs coated on quartz substrate (a) without, and (b) with thermal radiation treatment in N_2_O plasma. **Figure S3.** PL spectra of the untreated In_2_O_3_ NPs, thermal radiation treated In_2_O_3_ NPs for 7 and 10 minutes. **Figure S4.** HRTEM micrographs of the In_2_O_3_ nanocrystals with different facets ranging from (a) 3, (b) 4 to (c) 5 facets observed in the nanostructured In_2_O_3_ films. **Figure S5.** Tauc plots of (αE)^2^ against E for the In_2_O_3_ NPs and nanostructured In_2_O_3_ films. **Figure S6.** Planar view FESEM images of the In_2_O_3_ NPs deposited on quartz substrate (a) without, and (b and c) with thermal radiation treatment. Click here for file
