# Supplementary material for: Structure deformation of indium oxide from nanoparticles into nanostructured polycrystalline films by in situ thermal radiation treatment
Source: Nanoscale Res Lett. 2013 Oct 17;8(1):428. doi: 10.1186/1556-276X-8-428 (PMC3853438; doi:10.1186/1556-276X-8-428)
Supplement: Additional file 1 — Supplementary information. Figure S1. Schematic diagram and real time photographs of our home-built PA-HWCVD system. Figure S2. Photograph of the In2O3 NPs coated on quartz substrate (a) without, and (b) with thermal radiation treatment in N2O plasma. Figure S3. PL spectra of the untreated In2O3 NPs, thermal radiation treated In2O3 NPs for 7 and 10 minutes. Figure S4. HRTEM micrographs of the In2O3 nanocrystals with different facets ranging from (a) 3, (b) 4 to (c) 5 facets observed in the nanostructured In2O3 films. Figure S5. Tauc plots of (αE)2 against E for the In2O3 NPs and nanostructured In2O3 films. Figure S6. Planar view FESEM images of the In2O3 NPs deposited on quartz substrate (a) without, and (b and c) with thermal radiation treatment. [file 1556-276X-8-428-S1.docx]

Supplementary Information for

**Structure deformation of indium oxide from nanoparticles into nanostructured polycrystalline films by in-situ thermal radiation treatment**

Su Kong Chong^1*^, Azie Aniazizah^1^, Kee Wah Chan^1^, Hong-Quan Nguyen^2^, Wee Siong Chiu^1^, Chang Fu Dee^3^, Saadah Abdul Rahman^1^

^1^Low Dimensional Materials Research Centre, Department of Physics, University of Malaya, 50603 Kuala Lumpur, Malaysia.

^2^Department of Materials Science and Engineering, National Chiao Tung University, Hsinchu 30010, Taiwan.

^3^Institute of Microengineering and Nanoelectronics (IMEN), Universiti Kebangsaan Malaysia (UKM), Bangi, Selangor, Malaysia.

^*^Corresponding author: saadah@um.edu.my


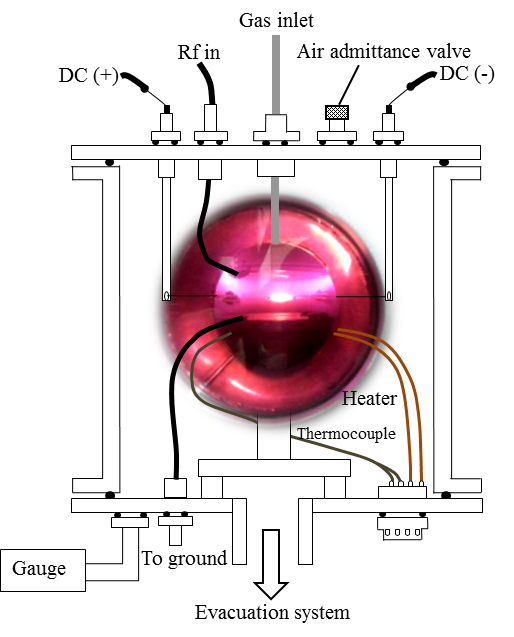


Figure S1 Schematic diagram and real time photographs of our home-built PA-HWCVD system.


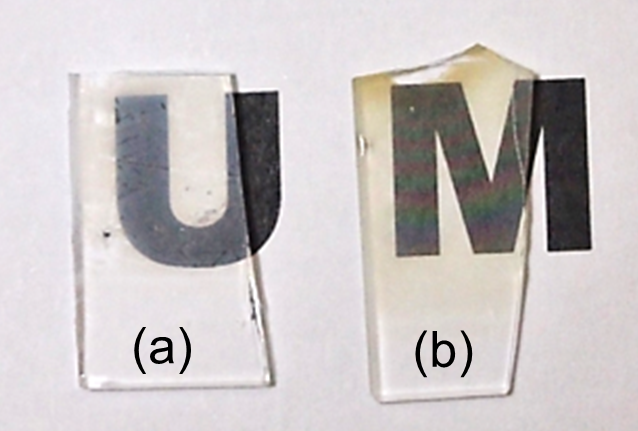


Figure S2 Photograph of the In_2_O_3_ NPs coated on quartz substrate (a) without, and (b) with thermal radiation treatment in N_2_O plasma.


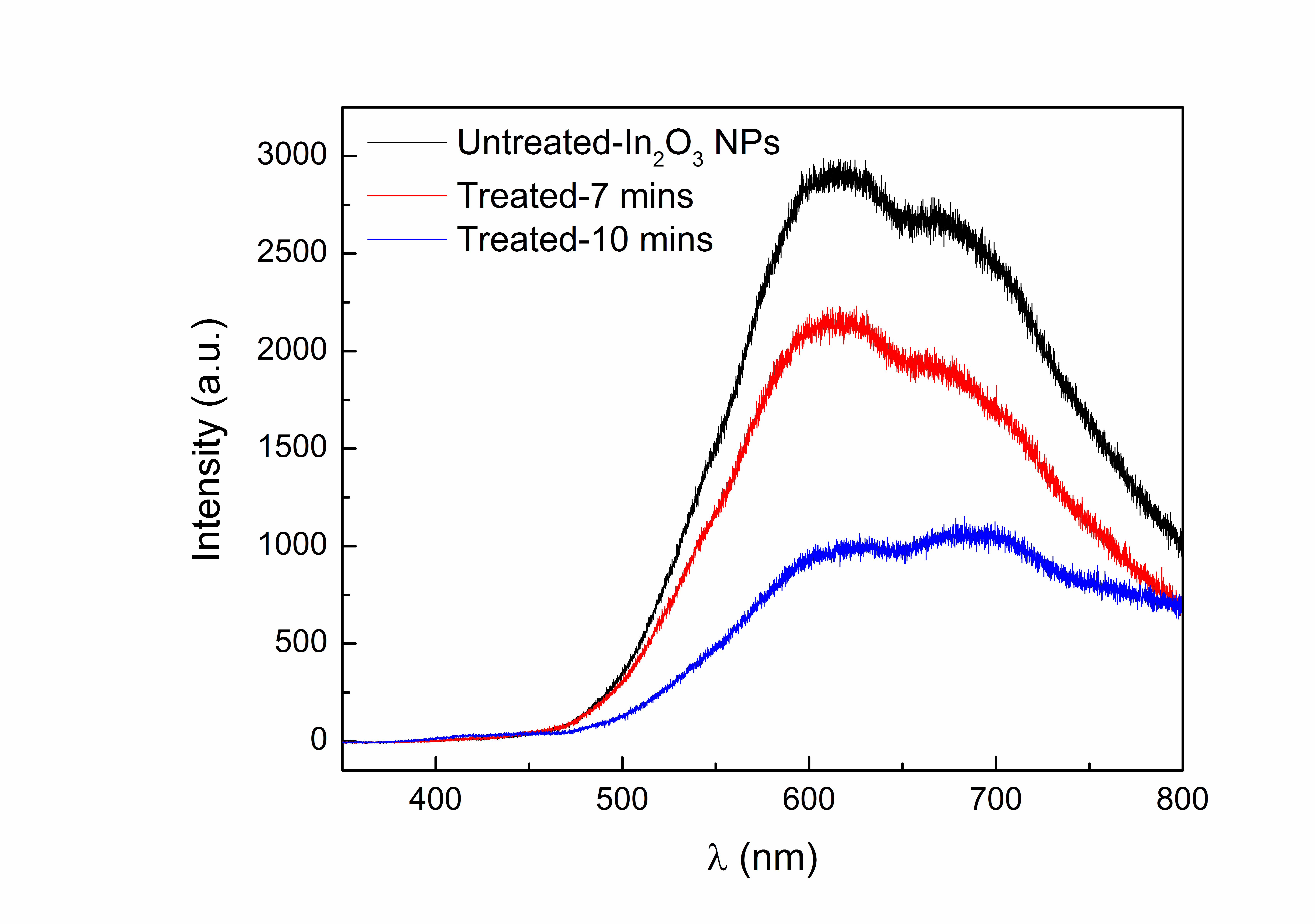


Figure S3 PL spectra of the untreated In_2_O_3_ NPs, thermal radiation treated In_2_O_3_ NPs for 7 and 10 minutes.


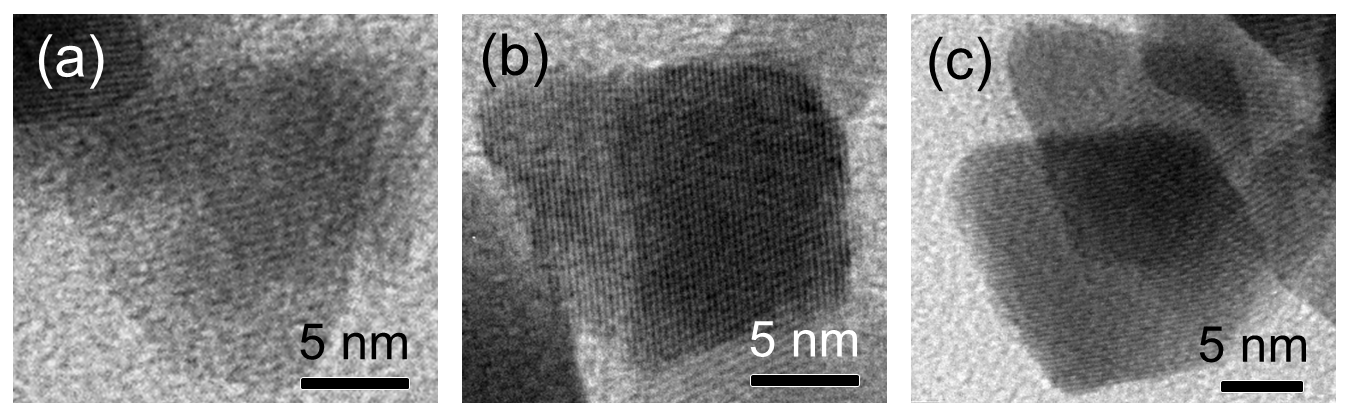


Figure S4 HRTEM micrographs of the In_2_O_3_ nanocrystals with different facets ranging from (a) 3, (b) 4 to (c) 5 facets observed in the nanostructured In_2_O_3_ films.


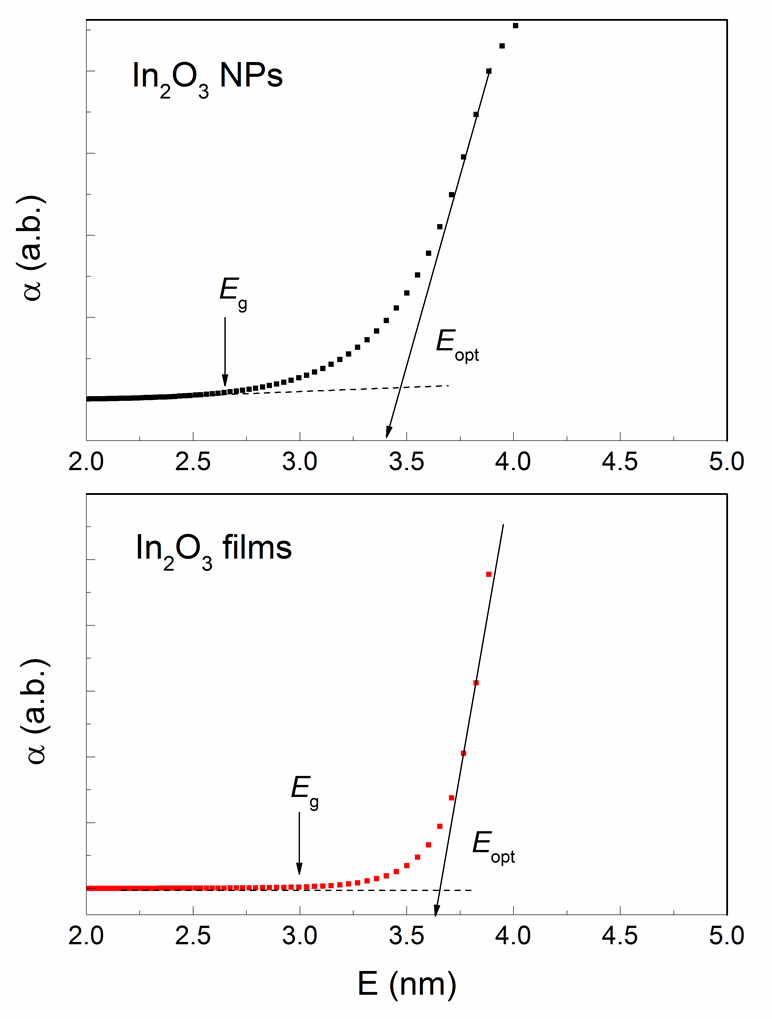


Figure S5 Tauc plots of (αE)^2^ against E for the In_2_O_3_ NPs and nanostructured In_2_O_3_ films.


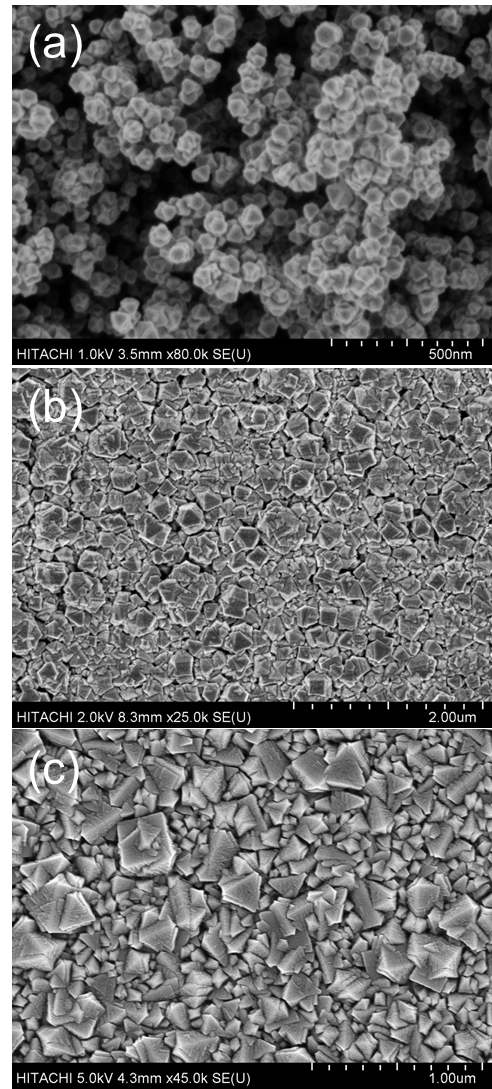


Figure S6 planar view FESEM images of the In_2_O_3_ NPs deposited on quartz substrate (a) without, and (b and c) with thermal radiation treatment. The surface morphology of the nanostructured In_2_O_3_ films at different treatment time of 7 and 10 minutes are shown in (b) and (c), respectively.
